# Overburden failure and water–sand mixture outburst conditions of weakly consolidated overlying strata in Dananhu No.7 coal mine

**DOI:** 10.1038/s41598-024-59240-y

**Published:** 2024-04-10

**Authors:** Jingzhong Zhu, Wenping Li, Bo Teng, Qinggang Lu, Dongding Li, Liangning Li

**Affiliations:** 1 School of Resources and Geosciences, China University of Mining and Technology, Xuzhou, 221116 China; 2 China Coal Xinjiang Energy Co., Urumqi, 830000 China; 3 Dananhu No.7 Coal Mine, SDIC Hami Energy Development Limited Liability Company, Hami, 839000 China

**Keywords:** Weakly consolidated strata, Overburden failure, Water–sand mixture outburst, Critical hydraulic gradient, Environmental sciences, Hydrology, Natural hazards, Solid Earth sciences

## Abstract

This study presents a case of weakly consolidated strata developed in Dananhu No.7 coal mine. Using a combination of numerical simulation, field measurement comparison, and the critical hydraulic gradient criterion, we investigate the overburden failure and the risk possibility of water–sand mixture inrush during excavation. The following are the principal findings: (1) Weakly consolidated rocks have poor physical characteristics, particularly when they are mudded and disintegrated after encountering water, which may become a favorable source of water–sand inrush; (2) The water-conducting zone develops to a height of 160.5 m with a crack-mining ratio of 15.29 times, extending upward to Toutunhe Formation aquifer. The predictions are consistent with measurements in adjacent mines with similar geological conditions; (3) Cracks without larger subsidence are developed at the front edge of the mining direction, and some parallel stepped cracks behind the goaf could be easily observed. Ground subsidence along the goaf center finally displays a symmetrically wide-gentle U shape; (4) The critical hydraulic gradient of Toutunhe Formation aquifer, aquifer above 3# coal seam, and aquifer of 3#–7# coal seam in Xishanyao Formation is 1.314, 1.351, and 1.380, the actual value is 0.692, 2.089, and 7.418 accordingly. It is inferred water–sand mixture outburst will not occur in Toutunhe Formation aquifer, while the potential risk exists in the aquifers of Xishanyao Formation. Through drainage and depressurization projects, a water–sand mixture outburst accident does not occur during excavation. This study reveals the overburden failure characteristics and the initiation mechanism of water–sand inrush in weakly cemented strata, as well as the internal relationship between them, which provides new research ideas for safe operation in other mining areas with similar geological conditions. The research work has certain practical guiding significance.

## Introduction

Because the mining area in western China is in the continental arid and semi-arid climate region, the water resources are deficient, the ecological environment is extremely fragile, and coal mining has a great impact on the geological environment^1–3^. Xinjiang, Northern Shaanxi, Shendong, and other mining areas have large reserves of coal resources, high calorific value, shallow buried depth, simple geological structure, and other natural advantages of mining. They have become the main coal energy base of hundreds of millions of tons^4–6^. Currently, some mines in western China mainly exploit coal seams deposited in Mesozoic Jurassic and Cretaceous coal-bearing strata. However, relatively shorter sedimentation leads to poor consolidation of rock mass, and the strata have formed the characteristics of weak cementation, easy weathering, poor permeability, argillization, and disintegration in water^7–9^. From the microscopic point of view, the mineral structure and cementation modes of rock determine the physical and mechanical properties and failure mechanism^10–14^, which is the main reason for the differences from those of the strata in the central and eastern mining areas. According to the geological and hydrogeological conditions of the western mining area, high-intensity and large-scale underground mining may cause water–sand mixture inrush, roadway support problems caused by weakly consolidated surrounding rocks, hydraulic support crushing, ground subsidence, phreatic and confined water level decline, and many problems in other safety and environmental impact^15–17^. Therefore, it is significant for mine safety operations and regional ecological protection to study the failure and deformation of overlying strata with weak cementation and the condition of water–sand mixture inrush under the mining influence.

Based on previous research, some experts and scholars at home and abroad have carried out a lot of work on the weakly cemented strata. Horizontal wellbore stability in the weakly consolidated sandstone stratum was studied to investigate the influence of rock failure around the wellbore on sand production^18^. Likewise, structural changes of weak cemented sandstone reservoirs associated with Cretaceous deposits were examined to improve the yield of Kazakhstan’s oil fields^19^. Super thick unconsolidated strata from Upper Triassic to Lower Cretaceous succession in the Kong Karls Land archipelago were analyzed to establish an improved understanding of the Mesozoic basin evolution, particularly in the northern Barents Sea^20^. In terms of the rock’s physical and mechanical properties, the relationships between the rock’s physical and mechanical parameters and the buried depth of typical coal mines in the Xinjiang and Ordos mining areas are analyzed. The differences between the physical and mechanical properties of rocks in western China and those of rocks with the same lithology in the central and eastern regions are proposed^21^. Based on the analysis of the microstructure of Jurassic weakly cemented sedimentary strata in Shendong mining area, the mineral compositions of rocks were quantified, and the quantitative relationship between mineral compositions and rock’s physical and mechanical parameters was determined by regression analysis method^22^. Using numerical and similar simulation tests, it is concluded that the failure pattern of thick weakly cemented overburden is considered as a “beam-arch shell” pattern, and the failure boundary is arched^23,24^. The discrete element numerical simulation software PFC^3D^ is used to visually show the caving characteristics of overlying strata under different mining heights. Besides, a FLAC^3D^ fluid–solid coupling numerical model was constructed to quantitatively study the permeability evolution law of overlying strata during mining, and to explore the dynamic response law of overburden migration, permeability evolution, and mining technical parameters of weakly cemented strata to groundwater system^25^. The stability of weakly cemented aquiclude under mining in the Ehuobulake mine in Xinjiang was studied. The mechanical model of mining stability was constructed, and the influencing factors and criterion of instability were proposed^26^. The fractal geometry theory and discrete element numerical simulation were used to study the evolution law of the fracture network during the layered mining of thick coal seams in the western mining area. The fractal evolution of overburden fractures experienced four stages, and the self-similarity between its development and expansion was high^27^. Under the circumstances of multi-coal seam repeated mining, the influence of plastic failure zone and pore pressure in surrounding rock, the development law of overburden fracture, and evolution characteristics of the seepage field in the Yixin coal mine were determined^4^. The phenomenon of water–sand mixture outburst in weakly cemented strata also occurs occasionally, so it has attracted the attention of scholars and engineers^28^. The probability of water–sand inrush disasters under different mining thicknesses is analyzed from the aspects of the formation process of water–sand inrush channels and the water pressure change^29^. Based on the background of the Tarangaole coal mine in Dongsheng Coalfield, the lithological characteristics and stability evaluation of 3^–1^ coal seam roof were analyzed, and the risk zonation of water–sand inrush was completed. The mechanism and influencing factors of water–sand inrush disaster in weakly cemented thick sand-conglomerate with large mining depth were proposed ^30^. Active and passive prevention and control mechanisms are proposed for the water–sand inrush in the high-potential energy environment. It is considered that the rocks prone to disintegration and argillation will cause a water–sand mixture inrush under the combined disturbance of mining and high-confined water^31^. Besides, through theoretical analysis, physical and numerical simulation, in-situ monitoring, and other means, the ground subsidence of the ecologically fragile western mining area under repeated mining was predicted and analyzed. Ground subsidence, settlement coefficient, and separation development height of repeated mining were obtained, and the maximum ground subsidence model verified by actual production was established^32,33^.

Currently, although some work has been done on the study of weakly cemented sedimentary strata in western mining areas, most of the research is limited to either overburden failure or water–sand inrush, and two aspects are not considered comprehensively. Besides, the studies of water–sand mixture inrush in weakly consolidated strata are relatively few, most of them are focused on the water–sand inrush in Neogene loose strata. We select Dananhu No.7 coal mine with weakly consolidated strata as an example in this study and discuss the failure characteristics of overlying strata and water–sand mixture inrush conditions together. Through the prior investigation of overburden failure, the development height of water-conducting fracture has been first identified, and then the material source location and other inducing conditions of water–sand mixture inrush are investigated. This research explores the relationship between overburden failure and water–sand outburst mechanism, and effective treatment has been carried out to eliminate the risk of water–sand outburst hazards. The scientific findings and measurements can be also applied to other mining areas with similar weakly consolidated strata and can serve as a shred of strong evidence for the analysis of water–sand outbursts. Importantly, this study presents a clear direction for investigating the roots of water disaster occurrence and can realize the purpose of safe mining under confined water bodies.

## Geological setting of the study area

**Figure 1:**
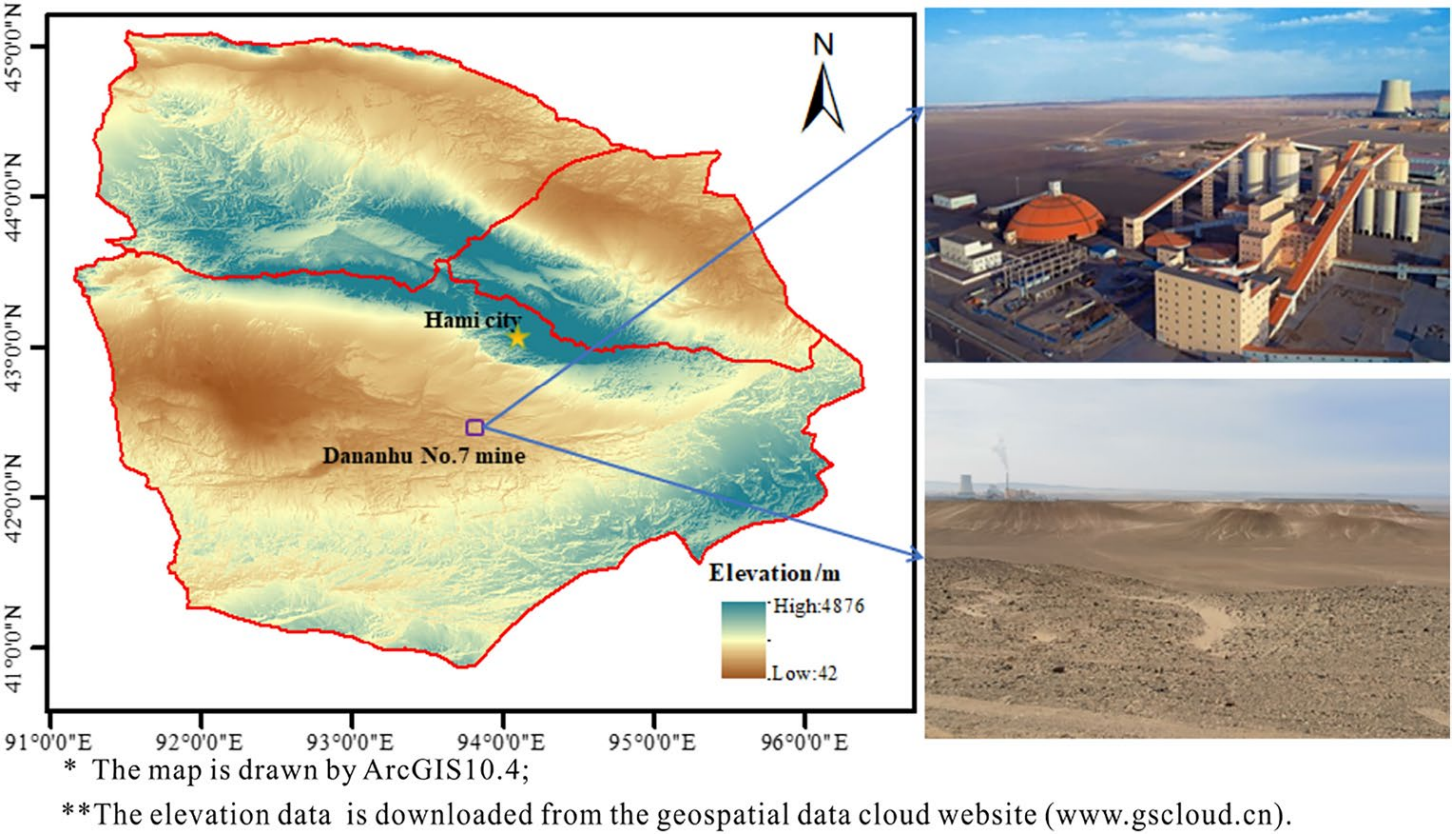
Location and geomorphology characteristics of the study area.

Dananhu No.7 coal mine in Hami City is situated in Nanhu town, 40.0 km southwest of Hami City. The geographical coordinates are 93°19′06″E–93°26′30″E and 42°22′30″N–42°24′30″N. The study area is located in the Gobi area, with less annual rainfall. There is no surface water system or other surface water bodies in and nearby areas, which is a desert area without surface vegetation (Fig. 1). The mine adopts the inclined shaft exploitation method. The coal mine is designed with five mining areas and three horizons. The preferred mining area is the first horizontal mining area. The three primary mining coal seams at the current time are 5# coal seam and 6# coal seam (due to a thin mudstone between 5# coal seam and 6# coal seam, combined mining of 5# coal seam and 6# coal seam) and 7# coal seam.

**Figure 2:**
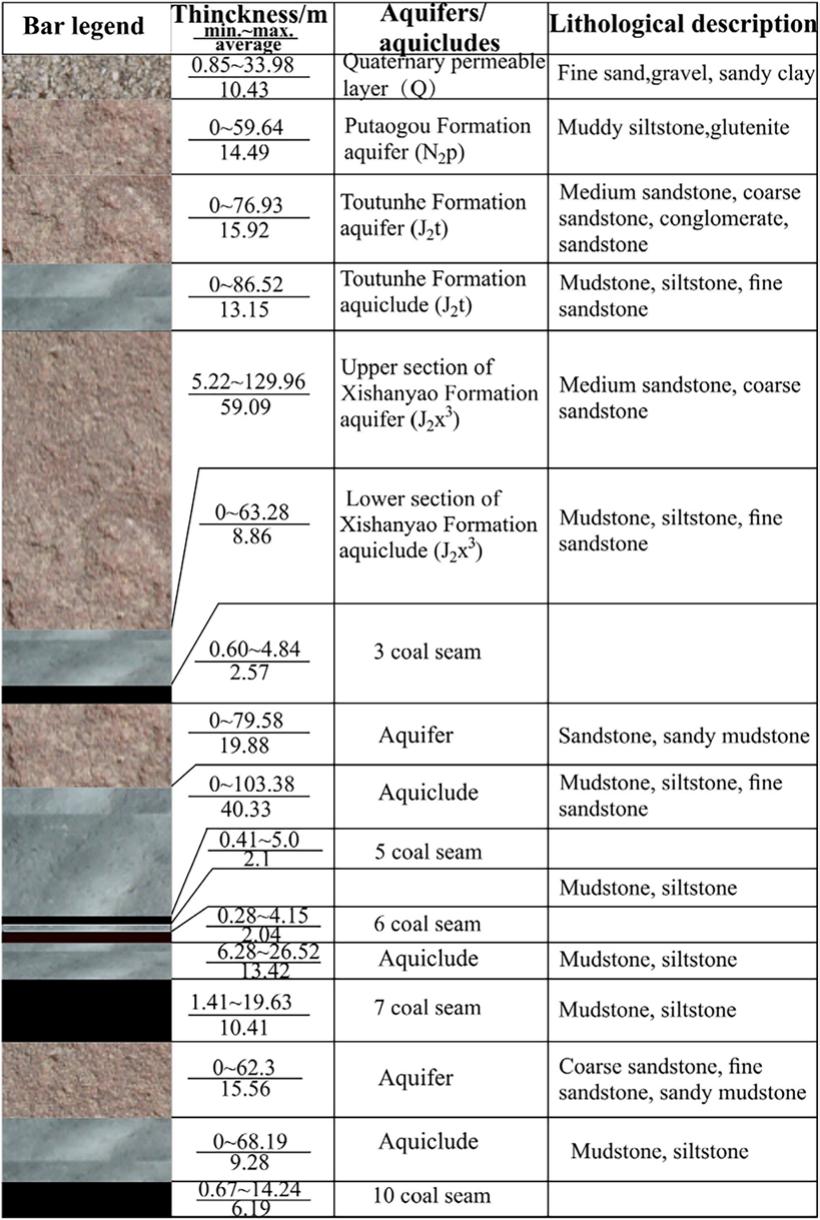
Comprehensive histogram of strata.

According to the actual geological drilling in the area, the strata are Quaternary (Q), Neogene Putaogou Formation (N_2_p), middle Jurassic Toutunhe Formation (J_2_t), Xishanyao Formation (J_2_x), and upper Carboniferous Wutongwozi Formation (C_2_wt), where the coal-bearing strata are Xishanyao Formation. As shown in Fig. 2. the aquifers comprise the Quaternary permeable sand layer, Putaogou Formation aquifer, Toutunhe Formation aquifer, and Xishanyao Formation aquifers including aquifer above 3# coal seam, aquifer of 3#–7# coal seam and aquifer of 7#–10# coal seam^34^. According to the borehole pumping tests, it is known that the water yield property of Putaogou Formation aquifer and Toutunhe Formation aquifer is extremely weak with the unit flow rate of 0.0009 L/(s·m), the water yield property of the aquifer above 3# coal seam is weak to medium with the unit flow rate of 0.0077–0.1207 L/(s·m), and the water yield property of the aquifer of 3#–7# coal seam and 7#–10# coal seam is weak^35^, with the unit flow rate of 0.0089–0.0758 L/(s·m) and 0.0332–0.0345 L/(s·m), separately. The aquicludes in the study area from top to down are Quaternary clay aquiclude, Toutunhe Formation sand-mudstone aquiclude, Upper Xishanyao Formation sand-mudstone aquiclude, sand-mudstone aquiclude above 7# coal seam and sand-mudstone aquiclude overlying 10# coal seam.

## Materials and methodology

### Sampling and experiment

**Figure 3:**
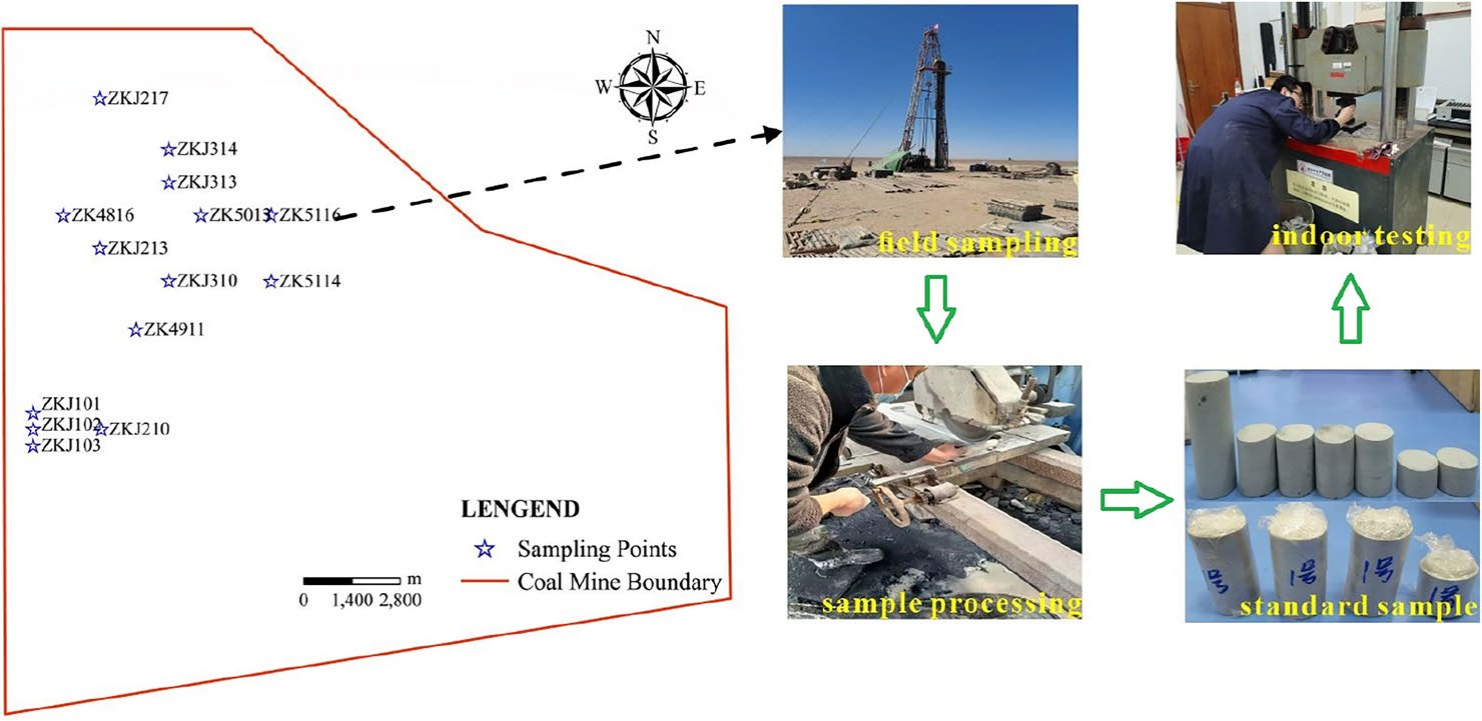
Sampling location, sample preparation and testing (Jingzhong Zhu and Dongding Li are involved in the process of field sampling, rock sample processing and testing, two authors are present in Fig. 3).

Fourteen boreholes were sampled when the ground survey drilling was implemented in the No.1 and No.3 mining areas. There were around two hundred core samples in total, and Fig. 3 illustrates the relative location of the sampling drilling. Since the size of on-site core samples cannot meet the testing requirements, it is necessary to further process the original samples into the standard samples before testing. Processing and sample size errors qualify for the recommended standards of the International Society of Rock Mechanics^36^. The testing was conducted in the State Key Laboratory of China University of Mining and Technology with a WES-D1000 electro-hydraulic servo universal testing machine. This equipment can be controlled by microcomputer and manual operation. The maximum testing force is 1000 kN, the span is 4–1000 kN, the minimum resolution is 0.01 kN, and ten sets of data can be recorded per second. Additionally, the deformation state of the sample under compression can be observed. Through the rock mechanics testing, the physical parameters such as compressive strength, tensile strength, shear strength, Poisson ratio, and elastic modulus were obtained, laying basic data for the subsequent theoretical research and analysis.

**Figure 4:**
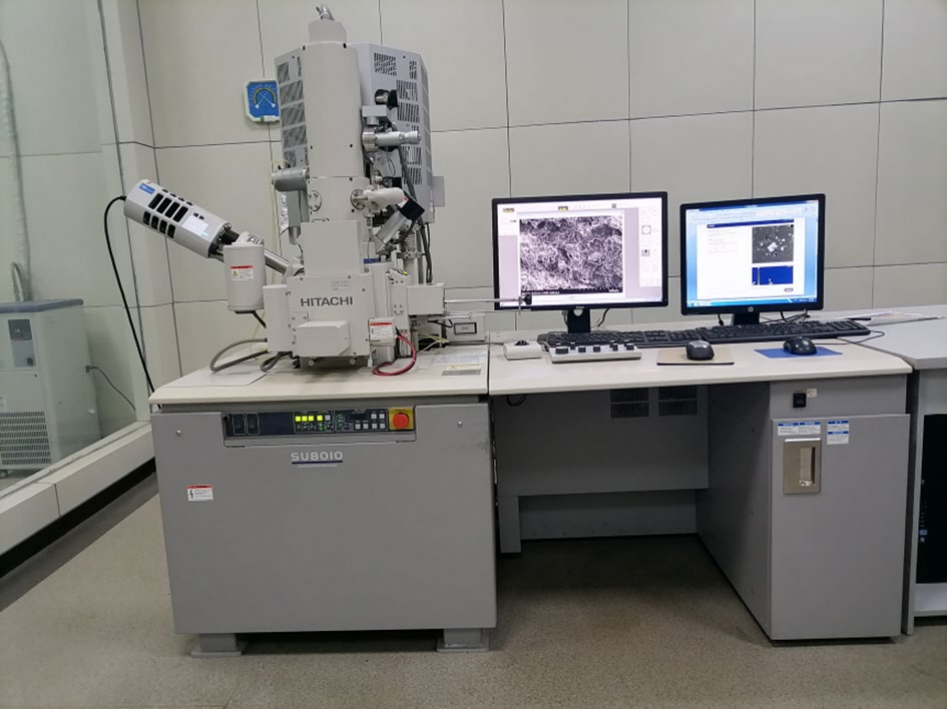
Scanning electron microscope (SEM) test device.

We also conducted the disintegration experiments of weakly cemented rock for sandstone and mudstone samples. According to the chemical testing of aquifer water, the water quality is generally characterized by high salinity and alkalinity. To present the actual interaction between rocks and water as much as possible, we placed samples into the prepared alkaline water. We observed the disintegration of rocks with different lithologies as time went by. The disintegration experiment results may serve as an explanation for materials source analysis of water–sand mixture inrush. Besides, we utilize the scanning electron microscope (SEM) to observe the microstructure with different lithologies, and we have a better understanding of the porous characteristics of rocks, and the microscopic testing process is shown in Fig. 4.

### Numerical simulation

Currently, the priority mining area is located at the No.1 mining area of the first level with the extraction of 6# coal seam and 7# coal seam, where the 11,701 working face is being mined. The 11,701 working face adopts the fully mechanized top coal caving and downward mining method, and the mining height is about 7.2 m. From the previous research and actual operation situation, we know when the coal seam is excavated, the aquifers in weakly cemented overburden are disturbed and destroyed, and the roof water–sand mixture inrush accident may occur^37–39^. Water-conducting fracture zone is often formed by overlying rock failure due to mining mineral resources. When the vertical fracture passes through the overlying confined aquifers and plenty of water–sand mixture sources and other conditions of water inrush are satisfied, water damage accidents will occur. To investigate the damage of weakly cemented strata and ground subsidence over the working face, the development characteristics of the water-conducting fracture zone and the risk prediction of water–sand inrush are preliminarily mastered.

In this study, COMSOL Multiphysics (CM) numerical simulation based on the finite element method is applied to study the overburden stress–strain, plastic failure zone, and surface subsidence during excavation. Based on the ground supplementary geological survey data of the mining area, the engineering geological model is simplified into six layers, which are Quaternary and Neogene strata, 6# coal seam overburden, 6# coal seam, 7# coal seam overburden, 7# coal seam, and 7# coal seam floor. The physical and mechanical parameters of each stratum are derived from this test and previous test data, as shown in Table 1.

**Table 1:** Mechanical parameters of rock strata for the simulation.

Layers category	Young modulus (GPa)	Poisson	Density (kg·m^−3^)	Cohesion (MPa)	Friction angle (°)	Thickness (m)
Quaternary and Neogene	3.02	0.33	2200	2.70	34.10	25.00
6# coal seam overburden	6.22	0.34	2580	3.70	33.20	182.00
6# coal seam	3.30	0.35	1350	2.10	36.00	5.00
7# coal seam overburden	4.08	0.35	2540	3.80	34.00	27.50
7# coal seam	3.60	0.34	1500	3.20	35.80	10.50
7# coal seam floor	6.14	0.35	2510	3.40	33.80	50.00

**Figure 5:**
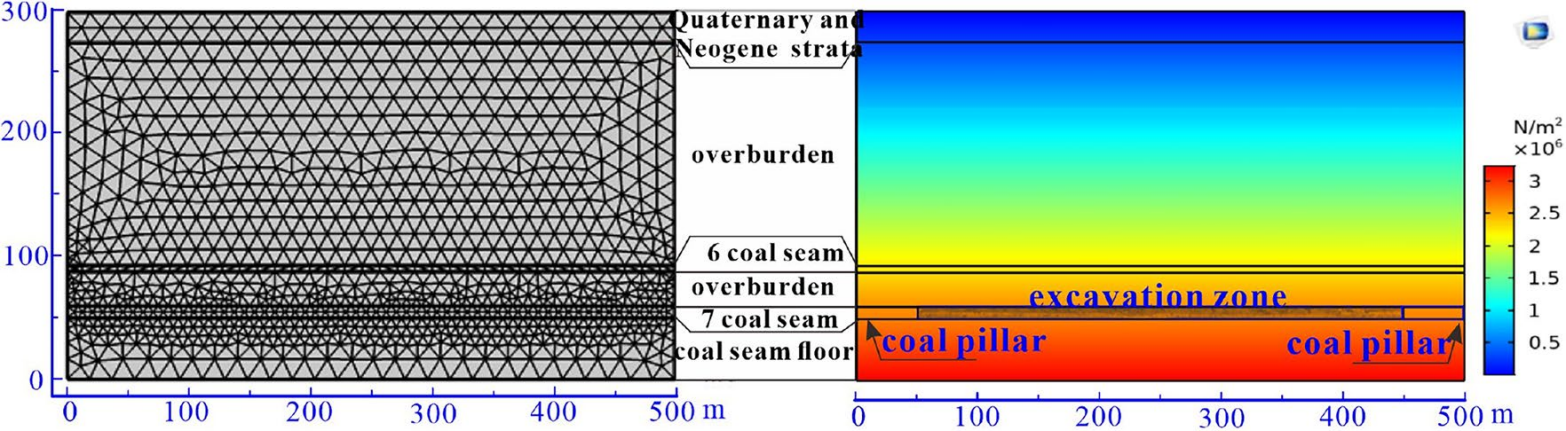
Meshing generation and initial stress field distribution.

The design size of the model is 300 m × 500 m, and the coal seam zone is finely meshed. The total number of grids is 4040, and the size of grids ranges from 0.0625 m to 18.5 m. Considering the influence of the mining boundary effect, 50 m protective coal pillars are reserved on the left and right boundaries, and the mining is step by step excavated from the left direction of the coal seam. The total excavation length is simulated to be 400 m. The mesh generation and distribution of the initial stress field before excavation is shown in Fig. 5.

## Results

### Physical and mechanical properties of weakly consolidated rock

**Figure 6:**
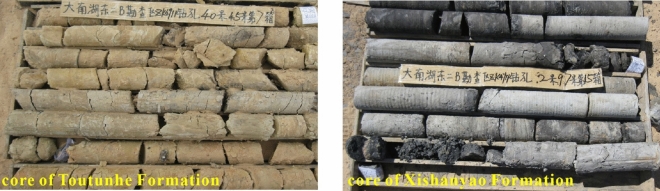
Cores of Toutunhe Formation and Xishanyao Formation (cores from ZK4911 borehole) (This image is taken by Jingzhong Zhu and Dongding Li during field sampling at Dananhu No.7 coal mine).

**Figure 7:**
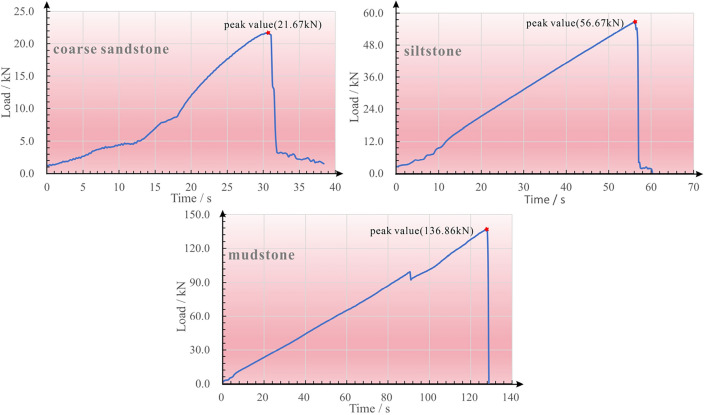
Load test curve of different lithology rocks.

In terms of field borehole sampling, the integrity of core samples in Xishanyao and Toutunhe Formation is poor and weakly consolidated as shown in Fig. 6, and the lithology of rock masses mainly consists of medium-coarse sandstone and mudstone. Besides, indoor uniaxial compressive strength tests are carried out on rocks of different lithologies, and the load test curve is shown in Fig. 7. Since the porosity of coarse sandstone is relatively large, the early compression is mainly pore compression, and the load growth is slow. When the pores of rocks are compacted, the compressive strength growth rate becomes larger, but the mechanical properties are generally poor. When the load increases to 21.67 kN, irreversible plastic failure occurs in the rock, and the compressive strength decreases rapidly, but there is still residual strength.

Compared with coarse sandstone, because the porosity of siltstone and mudstone is relatively smaller, the load shows a linear growth trend, the mechanical properties are better, and the ultimate load is 56.67 N and 136.86 kN, respectively. If the resisting-compressive limit is exceeded, the strength decay rate is larger and the residual strength is almost zero. Under the influence of mining, it is inferred that once the aquiclude with mudstone and siltstone is subjected to plastic damage, its water resistance will be lost, and the damage degree of rock mass will be more severe. Meanwhile, the water along the fissures will flow down to interact with mudstone and siltstone, which will carry plenty of mud and sand into the working face.

**Figure 8:**
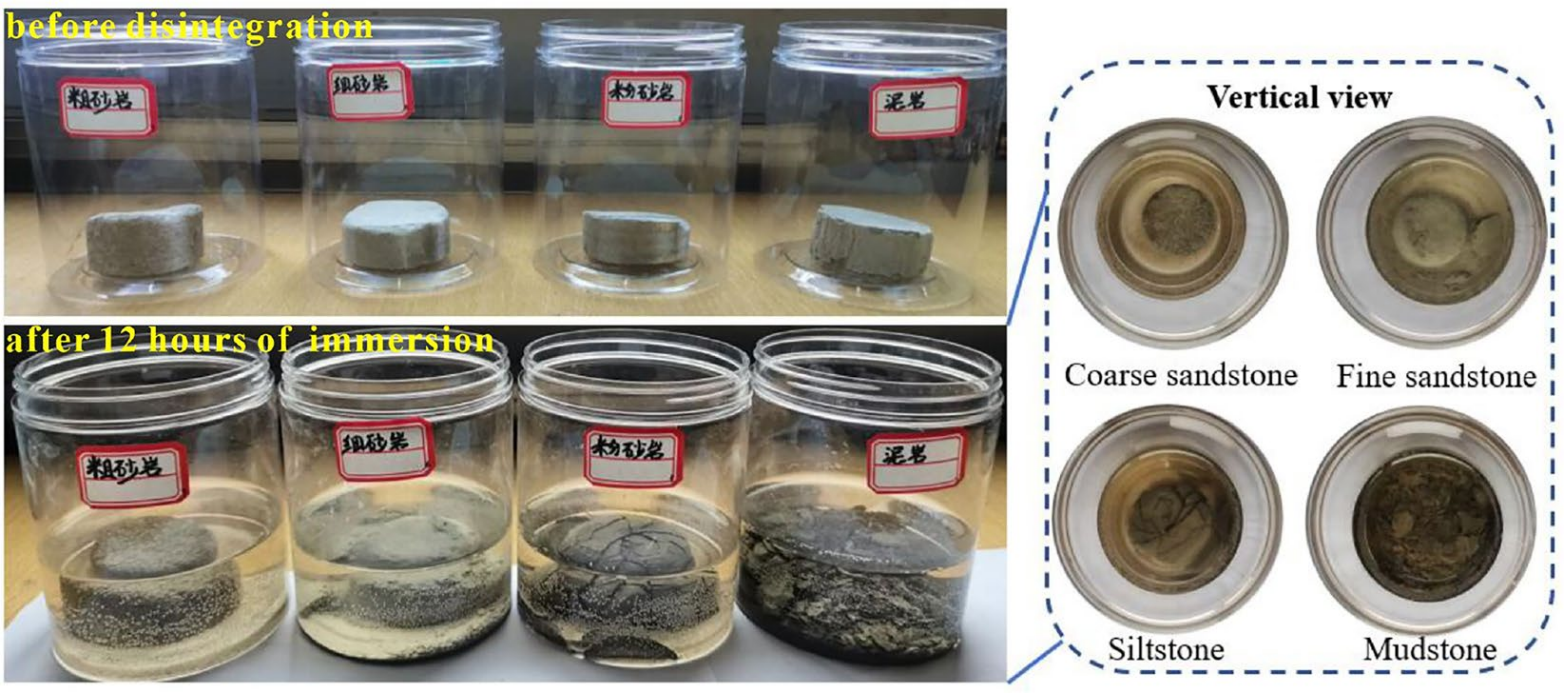
Disintegration test of weakly cemented sandstone and mudstone (This photo is taken by Jingzhong Zhu during the disintegration test conducted in the laboratory).

**Figure 9:**
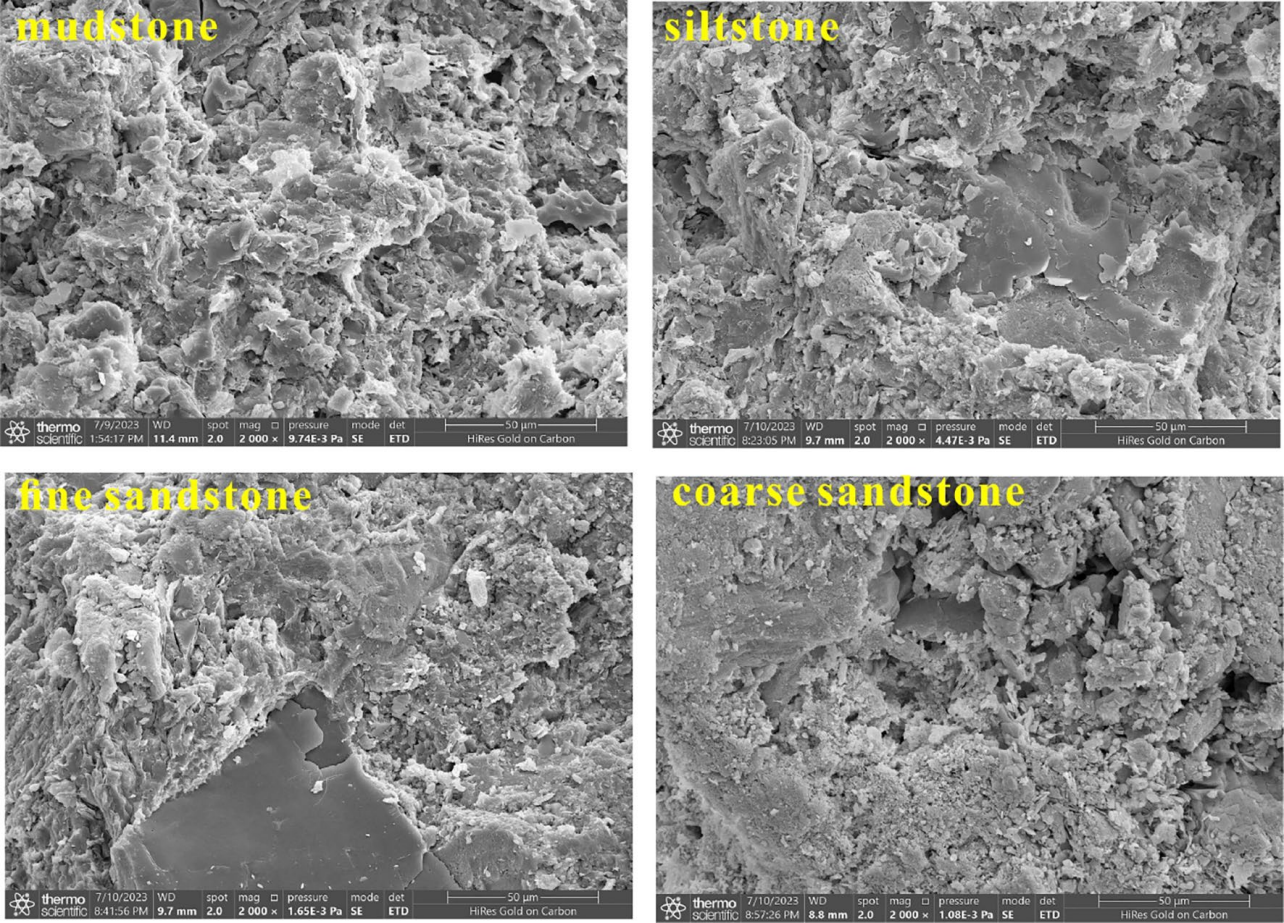
Microstructure of different lithology under SEM.

The rock masses disintegrate more easily when they are encountered by the alkaline water^40^. Through disintegration testing, we make a conclusion that mudstone, siltstone, and fine sandstone show varying degrees of disintegration ability, especially in mudstone and siltstone as shown in Fig. 8. Therefore, the weakly cemented sandstone and mudstone have the characteristics of water–sand inrush source. In addition, we observe the microstructure of rocks with various lithologies utilizing SEM test devices. As shown in Fig. 9, we easily find that the lamellar stacking structure is commonly developed in mudstone, this fact also in turn confirms the characteristics of the lamellar disintegration of mudstone. Meanwhile, the number and size of micro-pores in the rock gradually increase from mudstone to coarse sandstone. With the comprehensive influence of mining stress and high-pressure water, the natural pores become larger and a favorable channel for water–sand mixture inrush.

### Overburden deformation and failure

When the coal seam was excavated for 100 m, local stress concentration occurred in the coal pillar and the surrounding rocks of the working face. Under the complex action of rock self-weight and compressive shear stress, the immediate roof above the working face began to collapse, and the main roof appeared to transverse cracks. The plastic zone mainly developed in the range of 6# coal seam and below, and the height of plastic deformation is less than 40 m where the caving zone began. As the working face continued to advance to 200 m, the range of stress concentration area gradually expanded, and strata developed in 50 m above 6# coal seam were affected. The plastic deformation zone extended to 6# coal seam roof. This meant that the immediate roof of the working face completely collapsed. The extension scale of the transverse crack in the main roof increased with the increase of the excavation length, and the vertical displacement of the strata increased. Water-conducting fracture channels were distributed within 100 m above the working face.

**Figure 10:**
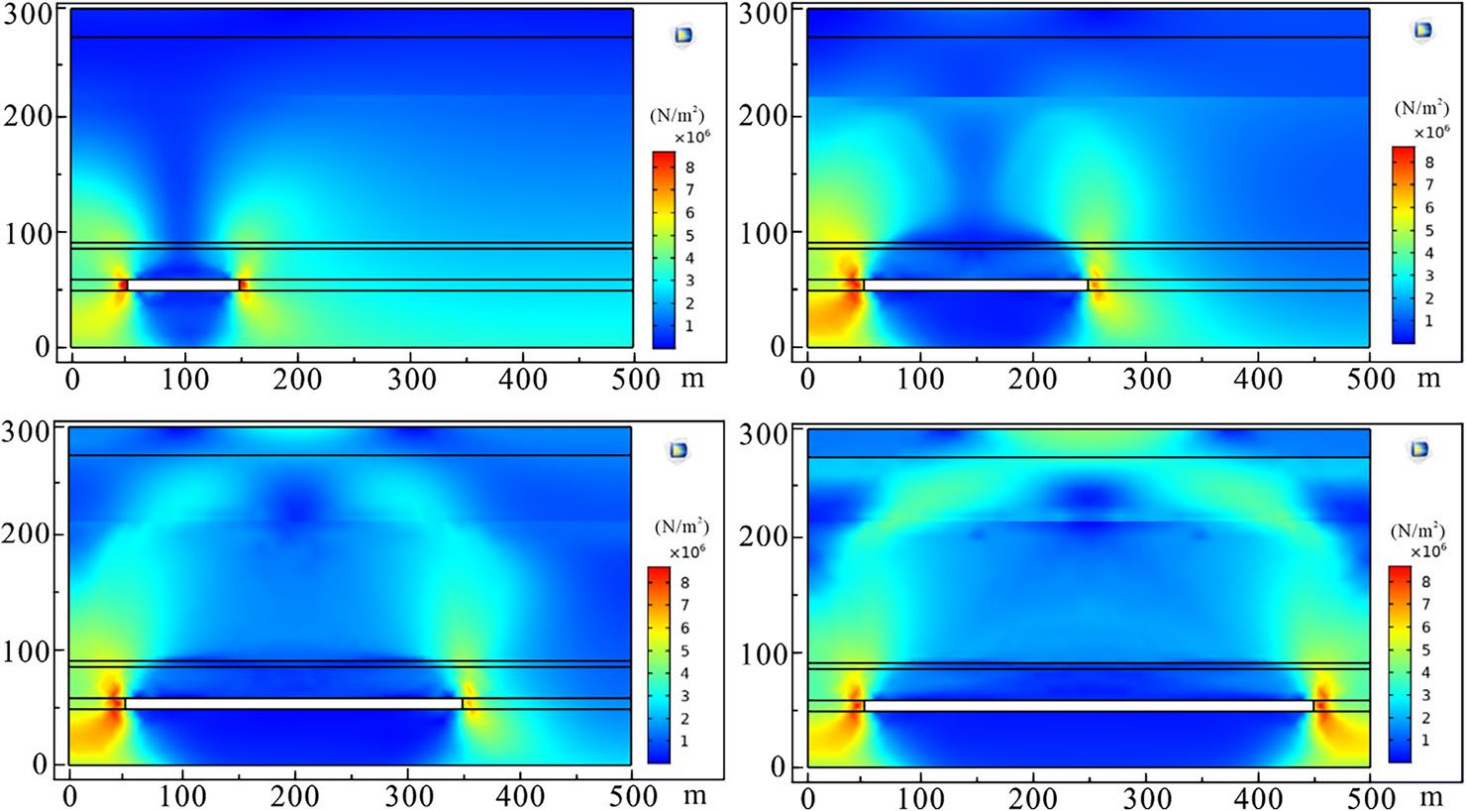
Stress field distribution at different mining stages (100 m, 200 m, 300 m and 400 m successively).

**Figure 11:**
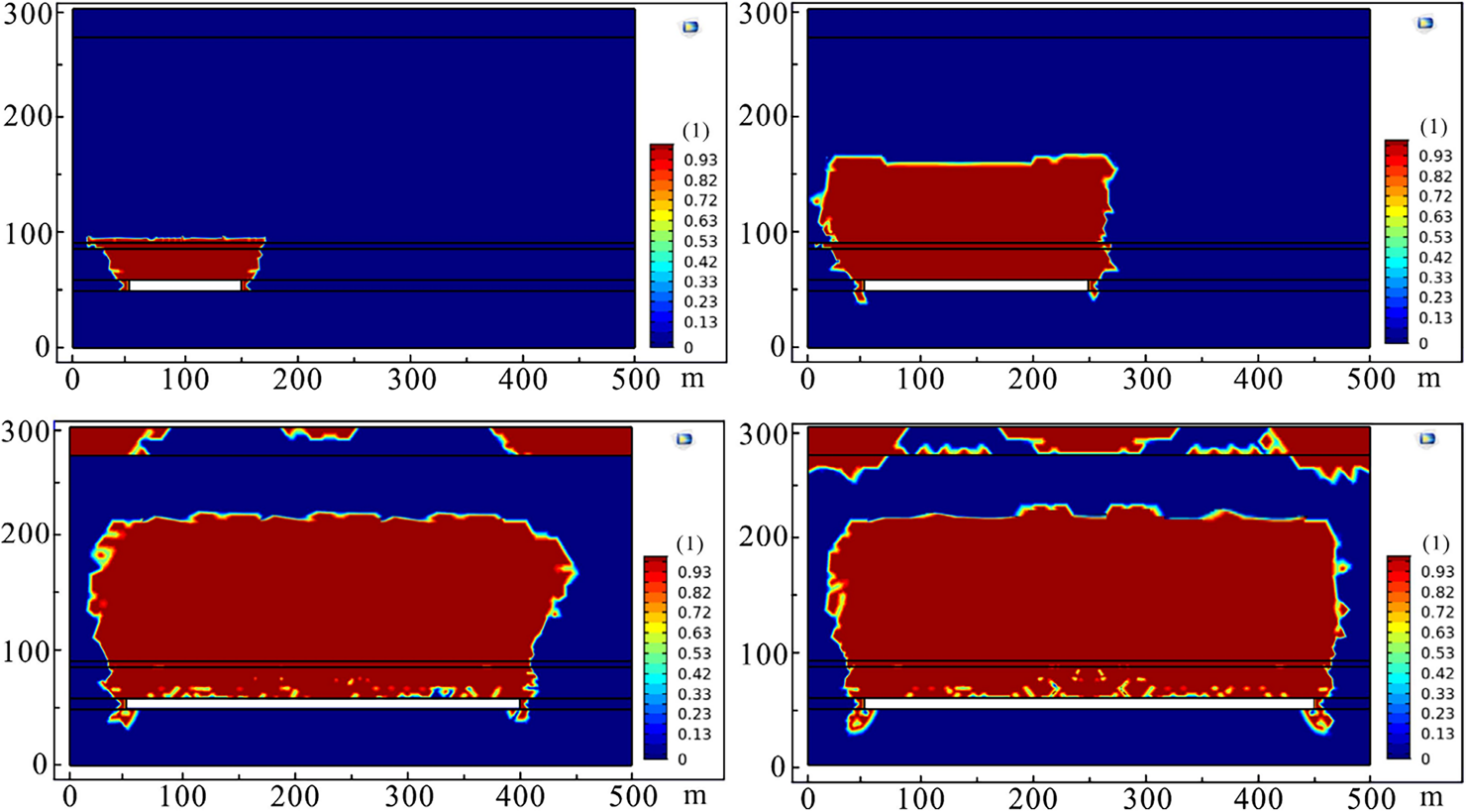
Plastic deformation at different mining stages (100 m, 200 m, 300 m, and 400 m successively).

After that, the working face continued to excavate 100 m, and the range affected by mining-induced concentrated stress further became larger. Due to exceeding the ultimate strength of rock mass, the obvious plastic zone occurred in the affected strata. Plastic deformation began to extend further upward, and the development height was about 150 m and its location was more than the aquifer above 3# coal seam of Xishanyao Formation. When the working face was fully extracted, that is, the working face was excavated to 400 m, and the concentrated stress scale of the strata in Toutunhe Formation and Xishanyao Formation below Neogene was the largest in the whole mining process. The stress field was symmetrically distributed along the center of the working face. 6# coal seam and overlying strata had obvious plastic failure, and the plastic failure zone developed to about 160.5 m above the working face. The stress distribution and plastic deformation of strata in different mining stages are shown in Figs. 10 and 11.

Based on the above analysis and research, we confirm that the development height of the caving zone is about 37.3 m, the height of the water-conducting fractured zone is about 160.5 m, namely the caving-mining ratio is 3.55 times and the crack-mining ratio is 15.29 times. Besides, the adjacent Dananhu No.1 coal mine, Dananhu No.5 coal mine, and Shajihai coal mine with similar geological conditions also conducted the “two zones” height measurement projects^41,42^, and the comparison results are shown in Table 2. Although some deviations between the predicted results and the actual results exist, the simulated results are approximate to the measured results of the coal mine on the whole, meaning that the simulation results are relatively reliable.

**Table 2:** Comparison results between simulated and measured “two belts”.

Coal mine	Mining thickness (m)	Caved height (m)	Fractured height (m)	Caving-mining ratio	Crack-mining ratio
Dananhu No.1 coal mine	6.30	20.89	94.00–98.00	3.32	14.92–15.56
Dananhu No.5 coal mine	3.00	14.50–15.70	58.20–60.30	4.83–5.23	19.40–20.10
Shajihai coal mine	6.00	78.52–81.54	16.32–16.86	2.72–2.81	13.09–13.59
Dananhu No.7 coal mine	10.50	35.30	160.50	3.36	15.24

### Water–sand mixture outburst conditions

As mentioned above, the overlying rock mass is damaged under the influence of mining, and the original aquiclude loses its waterproof ability. Besides, under the extra action of the high potential energy aquifer, the pressured water will carry mud and sand along the fracture channel into the working face, which poses a great threat to the mine operation and the on-site personnel’s safety^43^. Therefore, it is very necessary to analyze the water–sand mixture inrush resulting from mining. The occurrence of roof water–sand inrush accidents often requires the following conditions:(1) material sources: the sources of water–sand inrush are usually the Quaternary loose sand layer in Northern China coalfields, while the ancient weathering crust and weakly cemented sandstone and mudstone in the contact zone between the bottom of Cretaceous and the top of middle Jurassic (K_1_/J_2_) in Western China^44–46^; (2) bursting channel: the primary fractures including joints and beddings, water-conducting faults, poorly sealed boreholes, and mining-induced water-conducting fractures serve as flowing channels for water–sand inrush, among which mining-induced water-conducting fractures are the ideal and common channels causing disasters; (3) high potential hydrodynamic environment: water–sand inrush usually do not happen in the low-potential hydrostatic environment. The reason is that the actual hydraulic gradient does not reach the anti-permeability critical hydraulic gradient of sand-mud particles^47,48^. If sand-mud particles are in the high potential hydrodynamic environment, the critical hydraulic gradient value of particle flow is easily exceeded, and the likelihood of water–sand mixture inrush will significantly increase; (4) storage space: the water–sand mixture flowing along the channels finally converges in the working face or roadway. If the accident occurs in a closed blind roadway or goaf with a smaller space inhibiting the expansion of disasters, it is difficult to form a large-scale water–sand inrush.

**Figure 12:**
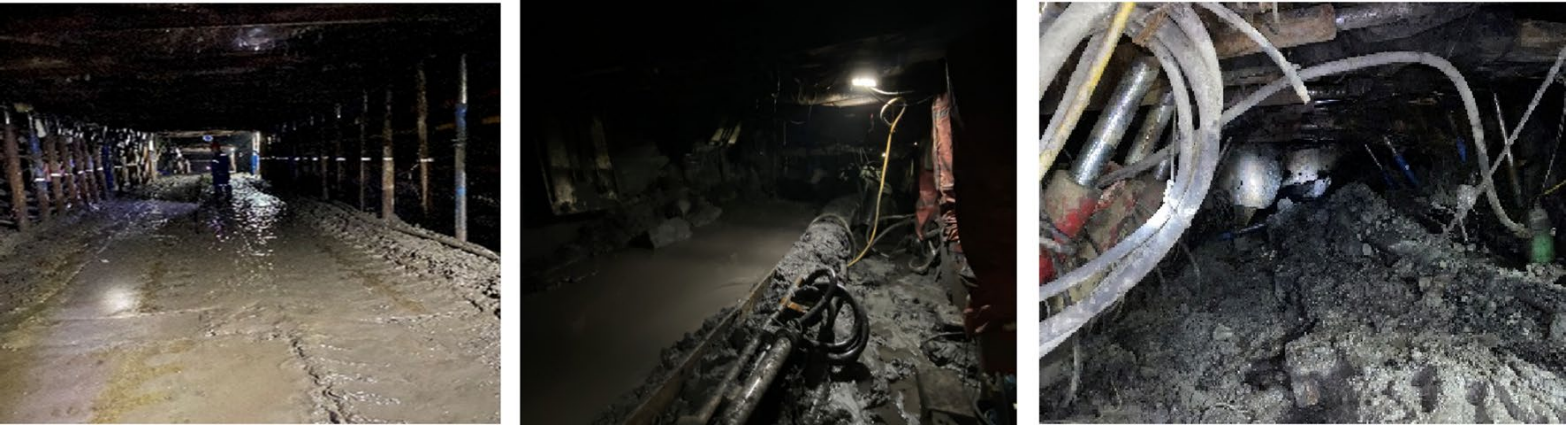
Roadway and open-off cut affected by water–sand inrush.

In the early mining, there was a small amount of water–sand inrush in the roof of the roadway and an open-off cut of the working face, as shown in Fig. 12. Furthermore, roof water aggravated the mudding process of floor stratum in the working face, and a larger amount of soften mudstone seriously restricted the hydraulic support propulsion. Accumulated water and sand in the local low-lying areas even destroyed the electrical equipment. Thus, it is necessary to investigate the cause and prevention of water–sand mixture inrush.

The geological structure of the study area is simple and no poorly sealed geological boreholes exist, so we exclude the possibility of water–sand inrush accidents caused by them. Based on the study of overburden failure, the damaged overlying strata are located in the aquifers of Toutunhe and Xishanyao Formation, while the loose strata of Quaternary and Neogene Putaogou Formation are not affected by mining. We calculate the hydraulic gradient of the damaged aquifers, and the critical hydraulic gradient (*J*_cr_) and actual hydraulic gradient (*J*) of the given aquifer are calculated as follows.

1 $$J_{cr} = G_{s} - 11 - n + 0.5n$$

2 $$J = \frac{{1.366\left( {2H - M} \right)}}{{2\pi r_{w} \left( {lgR - lgr_{w} } \right)}} \ge J_{cr}$$

where $G_{s}$ is the specific gravity of sand (kN·m^−3^); *n* is the porosity of sandstone; *H* is the aquifer head (m); *M* is the thickness of aquifer (m); *R* is the influence radius (m) under water–sand inrush; ${r}_{w}$ is the radius of the fracture channel (m). It is assumed that the water-conducting fracture channel is circular, and half of the fracture width is treated as the radius of the fracture channel.

Based on the tests of rock physical and mechanical properties, pumping tests, and aquifer water table observation, the index values of each calculation parameter of the above formula are obtained as shown in Table 3.

**Table 3:** Hydraulic gradient calculation parameters for aquifers.

Aquifers	Volume weight (KN·m^−3^)	Porosity	Head (m)	Thickness (m)	Fissure channel radius (m)	Influenced radius (m)
Toutunhe Formation aquifer	2.53	0.21	15.25	20.20	2.58	46.18
Xishanyao Formation aquifer (above 3 coal seam)	2.62	0.24	42.17	47.31	2.48	88.66
Xishanyao Formation aquifer (3–7 coal seam)	2.56	0.17	95.80	30.79	2.84	129.95

After the theoretical calculation, the critical hydraulic gradients of Toutunhe Formation aquifer, aquifer above 3# coal seam, and aquifer of 3#–7# coal seam are 1.314, 1.351, and 1.380, respectively, and the actual hydraulic gradients are 0.692, 2.089 and 7.418 accordingly. Therefore, although the mining-induced fractures have been developed in Toutunhe Formation aquifer, the actual hydraulic gradient is less than the critical value, so it is inferred that this aquifer does not result in water–sand inrush accidents. While actual hydraulic gradients of aquifers above 3# coal seam and 3#–7# coal seam are greater than their critical values, so we speculate if water–sand inrush occurs during mining, the main threatening roots come from Xishanyao Formation aquifers.

## Discussion

### Characteristics of ground subsidence

**Figure 13:**
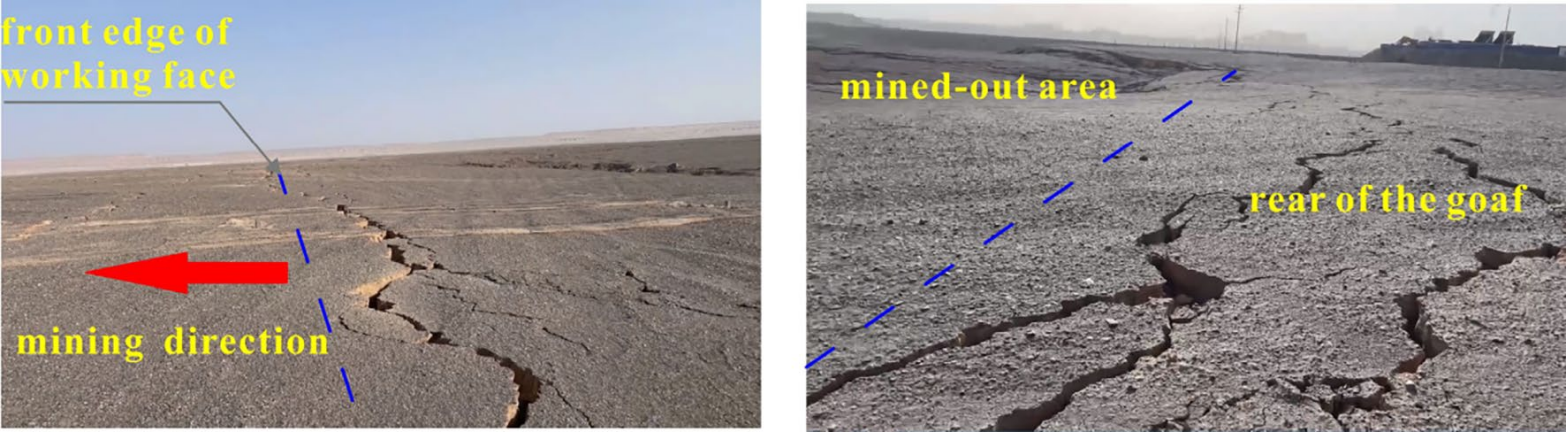
Post-mining ground surface fracture development (This photo is taken by Qinggang Lu at the subsidence area of 11701working face).

**Figure 14:**
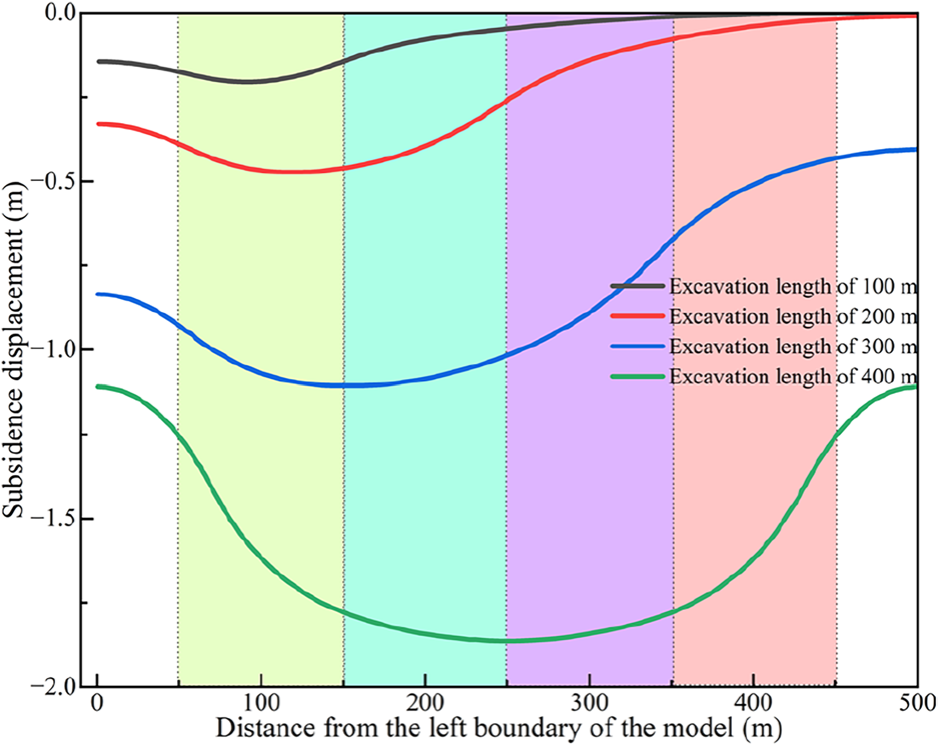
Subsidence characteristics of surface in different mining stages.

After coal seam excavation, the ground will show different degrees of subsidence^49,50^. During the operation of 11,701 working face, the geological department dynamically observes the ground subsidence caused by mining. As we can see from Fig. 13, there are different degrees of settlement cracks and cracks with no obvious subsidence begin to develop at the front edge of the working face advancement. Some parallel stepped cracks behind the goaf are found, these phenomena are in good agreement with the study of Ju and Xu^51^. Besides, we have set a monitoring line to dynamically investigate the vertical displacement of ground in the numerical simulation. The deflection of the ground surface at different mining stages is eventually simulated and predicted, as shown in Fig. 14. Compared with the actual measured value, the predictions are relatively smaller, which are basically consistent with previous findings from similar studies^52–54^.

In the initial mining stage, the subsidence displacement of the ground surface on both sides of the working face was relatively smaller, while the subsidence displacement along the goaf center was relatively larger than that of other areas. Besides, the overall deformation profile of the ground surface was asymmetrical. With the continuous advancement, the subsidence displacement of the ground surface gradually increased. When the working face advanced to 400 m, the maximum subsidence displacement of the ground was close to 2.0 m. The ground subsidence profile over the working face is different during mining. The ground subsidence is characterized by a symmetrical wide-gentle U shape along the goaf center when the working face is mined 400 m.

### Prevention measures of water–sand mixture outburst

According to the numerical simulation results, the development height of the water-conducting fractured zone is about 160.5 m where the coarse sandstone aquifer of Toutunhe Formation is developed. However, it can be seen from Table 3 that the critical hydraulic gradient is greater than the actual value, so water–sand mixture outburst accidents will not occur. The actual hydraulic gradient values (aquifer above 3# coal seam and aquifer of 3#–7# coal seam) are greater than the critical hydraulic gradient. Although the water yield property of the aquifer of 3#–7# coal seam is relatively weaker through hydrogeological surveys, considering that it has a high potential energy head, the potential risk of water–sand inrush is relatively higher, and the working face is bound to be threatened by water–sand inrush disasters.

Additionally, the special engineering geological conditions in the study area, namely the short deposition period of the strata result in poor consolidation of the rock, higher porosity, and stronger water sensitivity, which have been confirmed in this study and previous studies^12,55^. Besides, mining disturbance accelerates the water–rock interaction, and the mudding products of weakly cemented rocks become a favorable source of water–sand inrush. Thus, once these disaster-causing conditions are all available, the occurrence of mine water hazards is inevitable.

**Figure 15:**
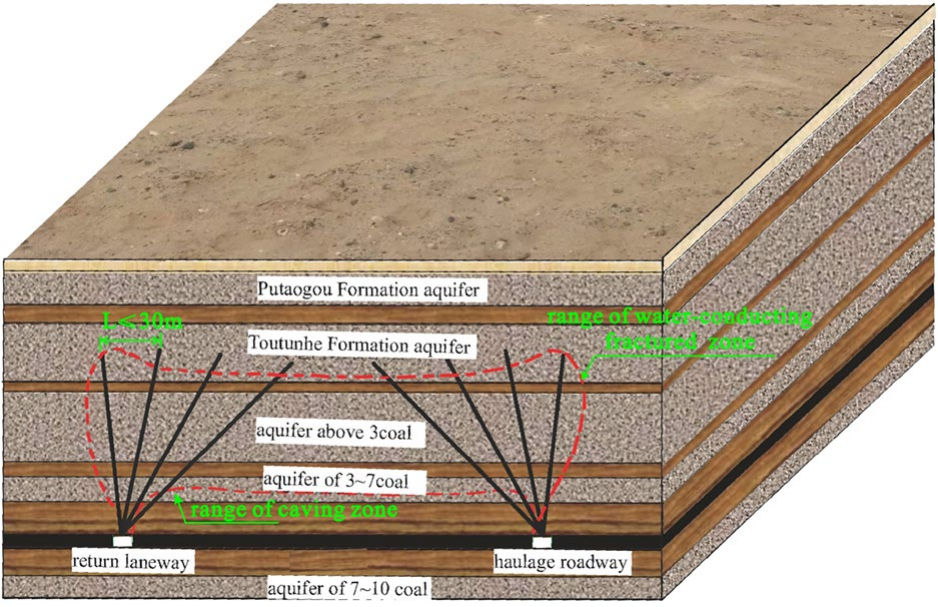
Layout of coalface roadway drainage depressurization boreholes.

To eliminate the potential risk of water–sand mixture inrush, multiple sets of roof drainage boreholes are constructed in the return laneway and haulage roadway of the 11,701 working face and the subsequent 11,601 working face. Considering the development location of the water-conducting fractured zone, the basic principle of borehole design parameters is that the final position of drainage boreholes extends to the aquifer of Toutunhe Formation, and the spacing of adjacent boreholes in the same group does not exceed 30 m as shown in Fig. 15. Through the continuous drainage of the target aquifers, volume of water output or water-drip in the working face roof become smaller than before, the water pressure of aquifers has fallen below range of the critical safety value. In terms of the 11,701 working face, the mining of the working face ended in December 2023, and the water outflow is maintained at 6.0–25.0 m^3^/h with an average volume of 17.79 m^3^/h. Until the termination of mining, there have been no water–sand mixture inrush accidents in the working face after the implementation of prevention measurements. The engineering practice confirms that the prevention and control effect of water disasters is better.

## Conclusions

Based on the analysis of hydrogeological conditions of Dananhu No.7 coal mine, we carried out physical tests and a numerical simulation to study the failure deformation characteristics of weakly consolidated overburden and ground surface subsidence characteristics under shallow coal seam mining. Additionally, theoretical analysis is used to analyze the potential risk of water–sand inrush in aquifers affected by mining, and active measures are taken for aquifers to transform the hydrodynamic environment. The main conclusions are as follows:

1. Using numerical simulation analysis, it is determined that the development height of the water-conducting fracture is about 160.5 m, and the crack-mining ratio is 15.29 times. Compared with the measured results of the adjacent coal mines, the predictions are in good agreement with the actual ones. Toutunhe Formation aquifer, the aquifer above 3# coal seam, and the aquifer of 3#–7# coal seam are affected by mining disturbance.
2. During the mining period, surface cracks with different subsidence appeared, and cracks without obvious subsidence were found at the front edge of the mining direction. Besides, parallel stepped cracks occurred behind the goaf. In the early stage, the subsidence displacement of the ground was relatively smaller, and there was asymmetry on both sides of the goaf center. As continuously mining, the subsidence displacement gradually increased, and the final profile showed a symmetrically wide-gentle U shape along the goaf center.
3. The critical and actual hydraulic gradient values of the damaged aquifers are quantified through calculation. It is inferred that water–sand inrush will not happen in Toutunhe Formation aquifer, while the potential risk in Xishanyao Formation aquifer still exists. Besides, the damaged weakly consolidated rocks will be mudded and disintegrated under the water–rock interaction, and sand-mud particles become a favorable source of water–sand inrush. Given the safety risk issues, the coal mine adopted the construction of roof drainage and depressurization boreholes. The effect of water disaster treatment is good through the validation of engineering practice, and the safe risk of water–sand inrush has been eliminated.

The relationship between the failure of overlying strata and the mechanism of water–sand mixture inrush is fully revealed in this study. The selected engineering case represents a kind of mining areas with weakly cemented strata, and because overburden failure characteristics and the start-up conditions of water–sand mixture inrush are similar to some extent in these mining areas. The demonstration case can provide an effective solution to the problem of overburden failure and water–sand mixture inrush in weakly cemented strata, aiming to achieve safe production under confined water bodies.

## Data Availability

The data supporting the findings of this article is included within the article.
